# Stroke severity and other predictors of venous thromboembolism in stroke patients—a population-based cohort study

**DOI:** 10.1016/j.rpth.2025.103220

**Published:** 2025-10-10

**Authors:** Ditte Vestergaard Hansen, Nick van Es, Henrik Toft Sørensen, Jonathan M. Coutinho, Nils Skajaa

**Affiliations:** 1 Department of Clinical Epidemiology, Aarhus University and Aarhus University Hospital, Aarhus, Denmark; 2 Department of Vascular Medicine, Amsterdam University Medical Center, Location University of Amsterdam, Amsterdam, the Netherlands; 3 Department of Neurology, Amsterdam University Medical Center, Location University of Amsterdam, Amsterdam, the Netherlands

**Keywords:** clinical predictors, cohort study, intracerebral hemorrhage, ischemic stroke, population-based, venous thromboembolism

## Abstract

**Background:**

Venous thromboembolism (VTE) frequently occurs after stroke, particularly within the first 3 months after diagnosis. Poststroke VTE is associated with increased mortality. Stroke severity is a known predictor of adverse prognosis; however, its ability to predict VTE is unclear. Knowledge regarding other predictors of poststroke VTE is lacking.

**Objectives:**

To identify clinical predictors of poststroke VTE in the subacute and acute phases, and to assess the association between stroke severity and poststroke VTE.

**Methods:**

In a population-based cohort study, we identified patients (aged ≥18 years and without recent VTE) with first-time ischemic stroke (*N* = 129,345) or intracerebral hemorrhage (*N* = 16,887) from 2004-2021. We computed the cumulative incidence proportion and subdistribution hazard ratio for VTE for each potential predictor in the subacute phase (3 months) and acute phase (7 days). For stroke severity, measured with the Scandinavian Stroke Scale, we calculated adjusted subdistribution hazard ratios in 2 multivariable models including (1) age and sex, and (2) predictors identified in the univariable analyses.

**Results:**

In the subacute phase, 1016 (0.8%) patients developed VTE after ischemic stroke, and 200 (1.2%) patients developed VTE after intracerebral hemorrhage. In univariable analyses, VTE risk was 2- to 5-fold higher in those with severe stroke, active cancer, and previous VTE. These findings were consistent for both stroke subtypes and follow-up periods. Multivariable analyses showed no substantial alterations in the estimates for stroke severity.

**Conclusion:**

Stroke severity, active cancer, and previous VTE strongly predict poststroke VTE in patients in the subacute and acute phases. These findings offer valuable insights and can be used to identify patients at elevated risk of VTE in whom extended thromboprophylaxis may be considered.

## Essentials

• Venous thromboembolism occurs after stroke and increases mortality, but predictors remain unclear.
• Cohort study of first-time stroke patients assessed predictors of venous thromboembolism.
• Severe stroke, active cancer, and prior venous thromboembolism predict poststroke venous thromboembolism.
• Findings highlight patients at higher risk who may benefit from extended thromboprophylaxis.

## 1 Introduction

Venous thromboembolism (VTE), a composite of deep vein thrombosis and pulmonary embolism, is a known complication of stroke, particularly during hospitalization and immediately thereafter [1,2]. Compared with the general population, patients with stroke have a 4- to 5-fold greater risk of VTE in the first 3 months after diagnosis, and poststroke VTE is associated with increased mortality [3,4]. Thromboprophylaxis with low molecular weight heparin is recommended for immobilized patients, regardless of their stroke subtype, and is typically discontinued after discharge [5].

Although findings from large randomized controlled studies do not support extended thromboprophylaxis in acutely ill hospitalized patients [[6], [7], [8], [9]], a recent meta-analysis focusing on the subgroup of patients with ischemic stroke within those studies found a clinically relevant 30% decrease in VTE with extension of thromboprophylaxis as long as 4 weeks after discharge [10]. Thus, identifying patients with stroke at higher risk of VTE after stroke may improve the risk-benefit ratio. However, little information on the clinical predictors of VTE in patients with stroke is currently available, and high-quality studies are lacking [4,[11], [12], [13], [14], [15], [16], [17]]. More than half of the existing studies had relatively small sample sizes (*N* < 600), and several studies did not report important information, such as the number of initial eligible participants or reasons for exclusion [11,[14], [15], [16], [17]]. Importantly, previous studies on stroke severity and VTE have either been inconsistent or have not investigated specific stroke subtypes, such as intracerebral hemorrhage [11,[14], [15], [16], [17], [18]]. Therefore, whether stroke severity is an important predictor of poststroke VTE remains unclear. Stroke severity is as a well-established predictor of other poststroke outcomes, including mortality, recovery, disability, mental health conditions, and workforce participation [[19], [20], [21], [22]].

In a Danish nationwide cohort study, we aimed to identify clinical predictors of poststroke VTE during both the subacute (3 months after diagnosis) and the acute (7 days after diagnosis) phases. Additionally, we evaluated the association between stroke severity and poststroke VTE.

## 2 Methods

### 2.1 Design and setting

We performed a cohort study using routinely collected data from Danish registries with nationwide, population-based coverage. The Danish healthcare system is universal and tax-supported and provides equal and free access to primary and hospital care, including acute stroke treatment [23]. Denmark has a rich array of population-based registries, which are linked at the individual patient level through the unique personal identification number assigned to all residents at birth or immigration [23]. In this study, we used data from the Danish Stroke Registry (Stroke Registry) [24], the Danish Civil Registration System [25], the Danish National Patient Registry (Patient Registry) [26], the Danish National Prescription Registry (Prescription Registry), and the Attainment Register [27,28]. Data sources are described in detail in Supplementary Table 1. Although the registries provide comprehensive coverage, certain limits exist. Of particular concern, medications administered during hospitalization, such as thromboprophylaxis relevant to stroke patients, are not captured. This limitation should be considered when interpreting our findings.

Ethical approval is not required for registry-based studies in Denmark. This study was approved by the Danish Data Protection Agency at Aarhus University (no. 2016-051-000001-1502). We followed the Strengthening the Reporting of Observational Studies in Epidemiology (STROBE) reporting guidelines [29].

### 2.2 Stroke management in Denmark

In Denmark, patients with suspected stroke are transported to specialized stroke units across the country and treated by a multidisciplinary team of healthcare professionals [30]. Effective prehospital and in-hospital management is in place in the country; ∼24% of patients with ischemic stroke receive reperfusion therapy, thrombolysis, or thrombectomy in Denmark, whereas the corresponding proportion in the United States is only 13% [31]. Clinicians are obligated to report stroke events and related clinical patient data to the Stroke Registry, a nationwide quality database with complete data from May 1, 2004 [24]. The sensitivity and positive predictive value of the coded diagnoses for stroke in this registry have been estimated to exceed 90% [32,33].

The national guidelines of the Danish Neurological Society recommend therapeutic anticoagulation (eg, low molecular weight heparin/unfractionated heparin or direct oral anticoagulant) for secondary prevention in patients with cardioembolic ischemic stroke (eg, in the presence of atrial fibrillation) [34], which is in agreement with the American Stroke Association and European Stroke Organisation guidelines for ischemic stroke [5,35]. For patients with noncardioembolic ischemic stroke, antiplatelet treatment (eg, aspirin) is recommended immediately, if they are not eligible for reperfusion therapy (thrombolysis or thrombectomy) [34]. For patients receiving reperfusion therapy, antiplatelet treatment is recommended, but administration is generally delayed until 24 hours after reperfusion therapy [36]. For patients with intracerebral hemorrhage, the optimal thromboprophylaxis remains complex and unclear because of concerns about worsening of intracerebral bleeding [[37], [38], [39]]. National and international guidelines emphasize that determining optimal treatment is challenging for clinicians, and the decision to initiate thromboprophylaxis is often evaluated on the individual patient level [40,41].

### 2.3 Study cohort

We identified Danish residents aged ≥18 years hospitalized with a first-time diagnosis of ischemic stroke or intracerebral hemorrhage between May 1, 2004 and December 31, 2021, as recorded in the Stroke Registry. The admission date of the first-time stroke event was classified as the index date. Patients with a history of VTE within 3 months before the index date were excluded to mitigate the risk of misclassification of recurrent events.

In the sensitivity analyses, study participants were excluded if they either had a hospital diagnosis of atrial fibrillation before the index date or had filled ≥1 prescriptions for an anticoagulant medication within 90 days before the index date. Additionally, study participants were censored if they filled a prescription for an anticoagulant medication during follow-up.

### 2.4 Stroke severity and other potential predictors

We assembled a list of 16 potential predictors according to previously constructed VTE prediction scores and clinical knowledge (detailed description of each potential predictor is provided in Supplementary Table 2) [11,42,43]. These predictors included stroke severity; active cancer; previous VTE; smoking; recent trauma or surgery; other comorbidities, such as heart failure, chronic kidney disease, chronic obstructive pulmonary disease, chronic inflammatory disease, and paralysis; postmenopausal hormone replacement therapy; Essen risk score; educational level; and cohabitation status. The Essen risk score is a well-known, simple clinical score used to predict recurrent ischemic stroke and other cardiovascular outcomes (detailed description is provided in Supplementary Table 3) [44]. Potential predictors were identified from records in the Stroke Registry, hospital-based diagnoses recorded in the Patient Registry, and filled prescriptions recorded in the Prescription Registry.

Specifically for stroke severity, we looked further into its role as an independent predictor given its applicability to all stroke patients. Several scales have been developed to quantify neurologic impairments after stroke, such as the Scandinavian Stroke Scale (SSS) [45]. The SSS is a validated neurologic scale conceptually similar to the National Institutes of Health Stroke Scale (NIHSS) and assessed in patients with stroke at admission to a stroke unit [46]. SSS scores range from 0 (worst) to 58 (best) [45]. We grouped strokes into mild (SSS: 43-58), moderate (SSS: 26-42), or severe (SSS: 0-25) categories, which have been shown to correspond well with conventional NIHSS groups (mild [NIHSS: 0-5], moderate [NIHSS: 6-14], and severe [NIHSS: 15-31]) [46]. Supplementary Table 4 provides a detailed description of the SSS.

### 2.5 VTE and follow-up

The outcome was VTE, defined as a composite endpoint of deep vein thrombosis in the lower extremities and pulmonary embolism. VTE events were identified from primary and secondary inpatient or outpatient discharge diagnoses recorded in the Patient Registry after the index date. Since the positive predictive value differs immensely between the emergency department and all other departments (31% and 75%, respectively), the emergency department diagnoses were ignored to avoid misclassification [47].

We created 2 follow-up periods with the aim of examining the risk of VTE in the acute and subacute settings. Starting from the index date, study participants contributed with follow-up for 3 months (subacute phase) and 7 days (acute phase), or until the date of VTE diagnosis, emigration, death, or December 31, 2021, whichever occurred first.

### 2.6 Statistical analyses

To assess potential predictors, univariable analyses with 95% CIs for each potential predictor were performed with the Aalen-Johansen estimator, to estimate cumulative incidence proportions, and the Fine-Gray regression, to estimate subdistribution hazard ratios (SHRs) [48]. Death from any cause was considered a competing event. To evaluate the association between stroke severity and poststroke VTE and whether stroke severity is an independent predictor, we calculated adjusted SHRs in 2 multivariable analyses including age and sex (model 1), and selected predictors identified from the univariable analyses (model 2). Selected predictors were chosen if the SHR was either >1.10 or <0.90, with a 95% CI not overlapping 1 [49]. All analyses were calculated for ischemic stroke and intracerebral hemorrhage separately as for the subacute (3 months) and acute (7 days) phases.

In a sensitivity analysis, we repeated the analyses among patients not actively using anticoagulant therapy. Missing data were handled under the assumption of missing completely at random by applying complete-case analysis, including only individuals with complete information on all variables [50].

## 3 Results

### 3.1 Baseline characteristics

We identified 129,345 patients with first-time ischemic stroke and 16,887 patients with first-time intracerebral hemorrhage during 2004-2021. Among the patients with ischemic stroke, 1016 developed VTE (0.8%) in the first 3 months (subacute phase) and 487 (0.4%) developed VTE in the first 7 days (acute phase); the corresponding numbers after intracerebral hemorrhage were 200 (1.2%) and 78 (0.5%), respectively. The median age was 72 years (IQR: 62-81), and 46% were women. A total of 71% and 40% of the strokes were mild in patients with ischemic stroke and patients with intracerebral hemorrhage, respectively. Other baseline characteristics of the study participants are shown in Table 1.

**Table 1:** Baseline characteristics of patients with ischemic stroke and intracerebral hemorrhage.

Characteristic	Ischemic stroke, *N* = 129,345	Intracerebral hemorrhage, *N* = 16,887
Age, median (IQR)	72 (62, 81)	73 (62, 82)
Age group, *n* (%)		
<65 y	38,482 (30%)	4865 (29%)
65-79 y	53,138 (41%)	6697 (40%)
≥80 y	37,725 (29%)	5325 (32%)
Sex, *n* (%)		
Female	59,487 (46%)	8108 (48%)
Male	69,858 (54%)	8779 (52%)
Stroke severity^a^, *n* (%)		
Mild	86,225 (71%)	6019 (40%)
Moderate	21,046 (17%)	3553 (24%)
Severe	14,480 (12%)	5507 (37%)
Unknown	7594	1808
Active cancer, *n* (%)	4712 (3.6%)	585 (3.5%)
Previous VTE, *n* (%)	4810 (3.7%)	646 (3.8%)
Smoking status, *n* (%)		
Current/occasional	38,736 (35%)	3235 (27%)
Former	32,216 (29%)	3618 (30%)
Never	40,349 (36%)	5297 (44%)
Unknown	18,044	4737
Trauma/fracture or surgery 8 wk before, *n* (%)	10,711 (8.3%)	1415 (8.4%)
Heart failure, *n* (%)	8362 (6.5%)	847 (5.0%)
Chronic kidney disease, *n* (%)	3419 (2.6%)	445 (2.6%)
COPD, *n* (%)	18,544 (14%)	2281 (14%)
Chronic inflammatory diseases, *n* (%)	7274 (5.6%)	875 (5.2%)
Paralysis, *n* (%)	314 (0.2%)	46 (0.3%)
Postmenopausal hormone replacement therapy, *n* (%)	5866 (4.5%)	786 (4.7%)
Essen stroke risk score, *n* (%)		
0	8,302 (6.4%)	1346 (8.0%)
1	20,220 (16%)	2841 (17%)
2	27,788 (21%)	3638 (22%)
3	32,180 (25%)	4208 (25%)
4	24,293 (19%)	3146 (19%)
5+	16,562 (13%)	1708 (10%)
Education level, *n* (%)		
High	17,267 (14%)	2558 (16%)
Low	54,329 (45%)	7026 (45%)
Medium	48,377 (40%)	6103 (39%)
Unknown	9372	1200
Cohabitation, *n* (%)		
Living alone	59,892 (46%)	7740 (46%)
Living with partner	69,135 (54%)	9105 (54%)
Unknown	318	42

COPD, chronic obstructive pulmonary disease; VTE, venous thromboembolism,

a According to Scandinavian Stroke Scale (SSS): Mild stroke (SSS: 43-58), moderate stroke (SSS: 26-42), and severe stroke (SSS: 0-25).

### 3.2 Predictors of VTE

**Figure 1:**
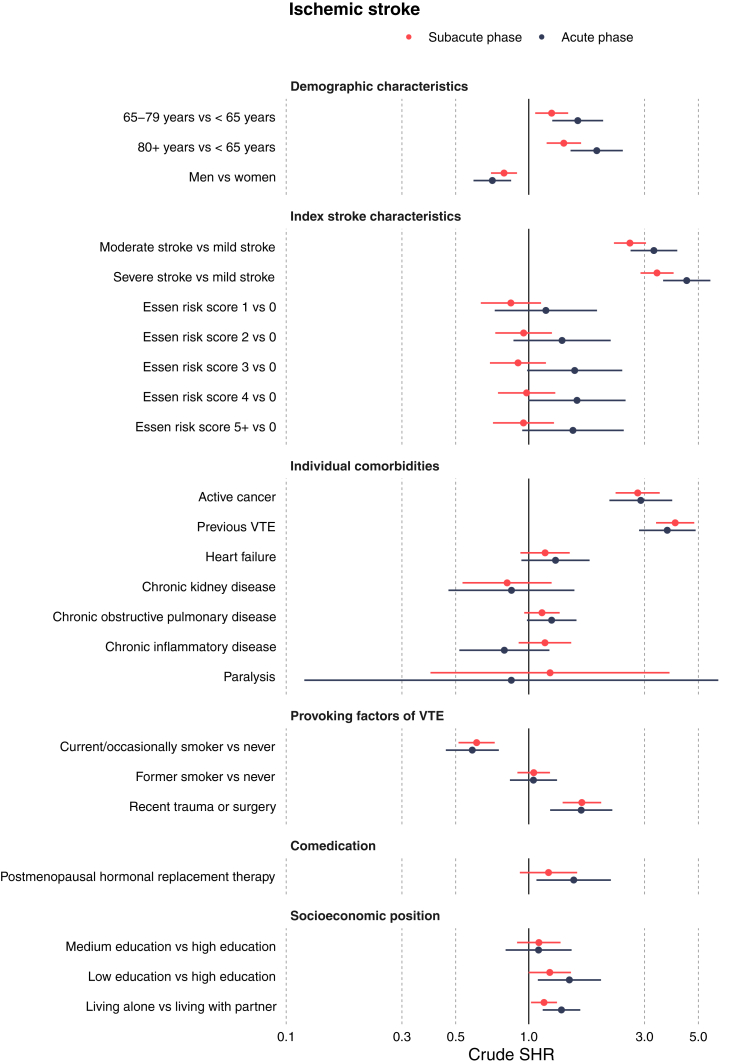
Forest plot with subdistribution hazard ratios for univariable analysis of potential predictors in ischemic stroke patients at subacute and acute phases. SHR, subdistribution hazard ratio; VTE, venous thromboembolism.

**Figure 2:**
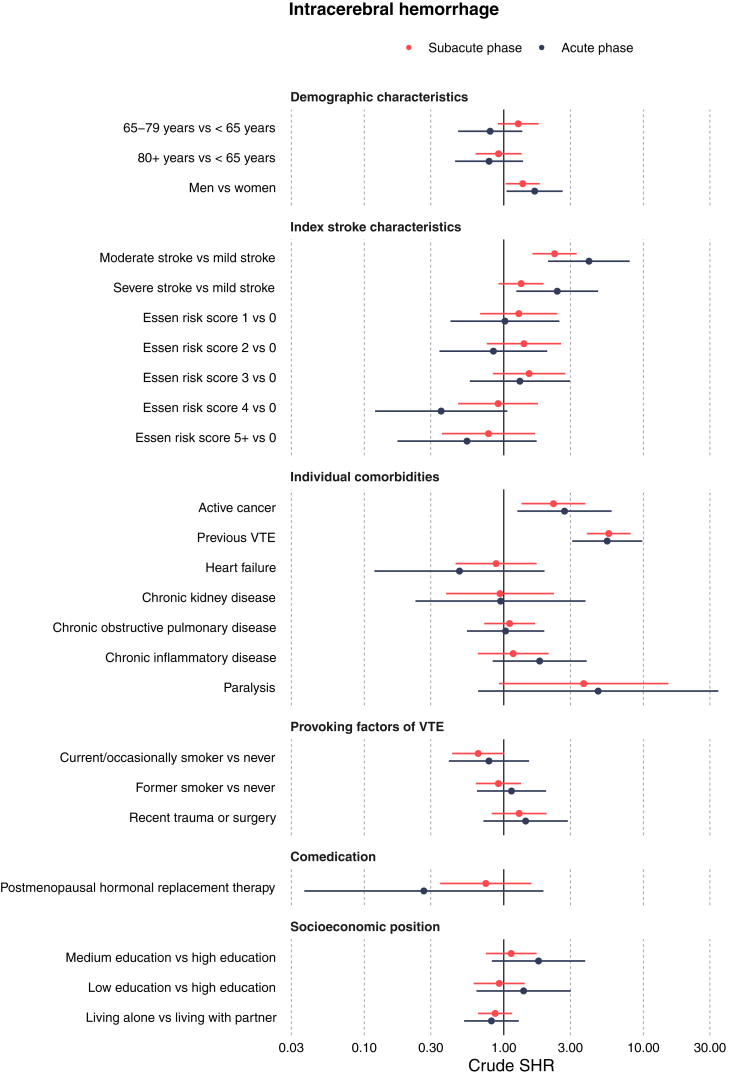
Forest plot with subdistribution hazard ratios in univariable analyses of potential predictors in patients with intracerebral hemorrhage, in subacute and acute phases. SHR, subdistribution hazard ratio; VTE, venous thromboembolism.

Figures 1 and 2 provide an overview of the SHRs for all potential predictors in the subacute and acute phases for ischemic stroke and for intracerebral hemorrhage. Overall, the cumulative incidence proportions for VTE ranged from 0.2% to 2.8% across the potential predictors after ischemic stroke and from 0.1% to 5.6% after intracerebral hemorrhage.

In patients with ischemic stroke, greater stroke severity was associated with a higher risk of VTE. The SHRs in the subacute phase were 2.61 (95% CI: 2.25, 3.04) for moderate stroke and 3.38 (95% CI: 2.89, 3.95) for severe stroke, compared with mild stroke. A similar pattern with an even greater risk increase was observed in the acute phase. Among patients with intracerebral hemorrhage, those with moderate stroke had the highest risk of VTE. The SHRs during the subacute phase were 2.31 (95% CI: 1.61, 3.33) for moderate stroke and 1.33 (95% CI: 0.92, 1.93) for severe stroke, compared with mild stroke. A similar pattern with elevated risk was present in the acute phase.

Active cancer was associated with an elevated risk of VTE after ischemic stroke, with an SHR of 2.81 (95% CI: 2.28, 3.46) in the subacute phase. Comparable risks were found in the acute phase for ischemic stroke as well as in both time periods for patients with intracerebral hemorrhage. The risk of VTE after ischemic stroke was 4-fold higher in individuals with a history of previous VTE in the subacute phase (SHR: 4.01; 95% CI: 3.35, 4.81), a finding consistent with the risk in the acute phase. Similarly, patients with intracerebral hemorrhage with and without a history of previous VTE had comparable risk. More details on the cumulative incidence proportions and SHRs for all potential predictors in the subacute phase and acute phase are shown in Table 2 and Supplementary Table 5, respectively.

**Table 2:** Risks and subdistribution hazard ratios for venous thromboembolism in the subacute phase for ischemic stroke and intracerebral hemorrhage.

Potential predictors	Ischemic stroke (*N* = 129,345)		Intracerebral hemorrhage (*N* = 16,887)	
	CIP, % (95% CI)	Crude SHR (95% CI)	CIP, % (95% CI)	Crude SHR (95% CI)
Stroke severity				
Mild	0.50 (0.45, 0.55)	Ref.	0.84 (0.60, 1.07)	Ref.
Moderate	1.30 (1.15, 1.45)	2.61 (2.25, 3.04)	1.92 (1.47, 2.38)	2.31 (1.61, 3.33)
Severe	1.70 (2.49, 1.91)	3.38 (2.89, 3.95)	1.15 (0.87, 1.43)	1.33 (0.92, 1.93)
Age				
<65 y	0.65 (0.57, 0.73)	Ref.	1.09 (0.80, 1.39)	Ref.
65-79 y	0.80 (0.73, 0.88)	1.24 (1.06, 1.45)	1.40 (1.11, 1.68)	1.27 (0.90, 1.78)
≥80 y	0.91 (0.81, 1.00)	1.40 (1.19, 1.64)	1.02 (0.75, 1.29)	0.92 (0.63, 1.34)
Sex				
Female	0.89 (0.81, 0.96)	Ref.	1.00 (0.79, 1.22)	Ref.
Male	0.70 (0.64, 0.76)	0.79 (0.70, 0.89)	1.36 (1.12, 1.60)	1.36 (1.03, 1.81)
Active cancer				
No	0.74 (0.69, 0.79)	Ref.	1.14 (0.98, 1.30)	Ref.
Yes	2.07 (1.66, 2.48)	2.81 (2.28, 3.46)	2.58 (1.29, 3.87)	2.27 (1.34, 3.84)
Previous VTE				
No	0.71 (0.66, 0.76)	Ref.	1.01 (0.86, 1.17)	Ref.
Yes	2.82 (2.35, 3.29)	4.01 (3.35, 4.81)	5.62 (3.84, 7.41)	5.65 (3.94, 8.10)
Smoking				
Never	0.88 (0.79, 0.97)	Ref.	1.36 (1.05, 1.68)	Ref.
Current/occasional	0.54 (0.36, 0.61)	0.61 (0.51, 0.72)	0.90 (0.57, 1.23)	0.66 (0.43, 1.01)
Former	0.92 (0.82, 1.02)	1.05 (0.90, 1.22)	1.25 (0.89, 1.61)	0.91 (0.63, 1.33)
Recent trauma, fracture, or surgery (8 wk before)				
No	0.75 (0.70, 0.80)	Ref.	1.16 (0.99, 1.33)	Ref.
Yes	1.24 (1.03, 1.45)	1.66 (1.38, 1.99)	1.49 (0.86, 2.12)	1.29 (0.82, 2.02)
Heart failure				
No	0.78 (0.73, 0.83)	Ref.	1.20 (1.03, 1.36)	Ref.
Yes	0.91 (0.71, 1.12)	1.17 (0.92, 1.47)	1.07 (0.38, 1.77)	0.88 (0.45, 1.72)
Chronic kidney disease				
No	0.79 (0.74, 0.84)	Ref.	1.19 (1.02, 1.36)	Ref.
Yes	0.65 (0.38, 0.92)	0.81 (0.53, 1.24)	1.13 (0.15, 2.12)	0.94 (0.39, 2.29)
Chronic obstructive pulmonary disease				
No	0.77 (0.72, 0.83)	Ref.	1.20 (1.03, 1.38)	Ref.
Yes	0.88 (0.74, 1.01)	1.13 (0.96, 1.34)	1.10 (0.67, 1.53)	0.91 (0.63, 1.33)
Chronic inflammatory disease				
No	0.78 (0.73, 0.83)	Ref.	1.18 (1.01, 1.35)	Ref.
Yes	0.91 (0.69, 1.13)	1.17 (0.91, 1.50)	1.38 (0.60, 2.15)	1.17 (0.65, 2.09)
Paralysis				
No	0.79 (0.74, 0.84)	Ref.	1.18 (1.02, 1.34)	Ref.
Yes	0.96 (0.00, 2.04)	1.22 (0.39, 3.80)	4.35 (0.00, 10.24)	3.74 (0.93, 15.12)
Postmenopausal hormone replacement therapy				
No	0.78 (0.73, 0.83)	Ref.	1.20 (1.03, 1.37)	Ref.
Yes	0.94 (0.69, 1.19)	1.21 (0.92, 1.58)	0.89 (0.23, 1.55)	0.74 (0.35, 1.58)
Essen stroke risk score				
0	0.85 (0.65, 1.04)	Ref.	0.97 (0.44, 1.49)	Ref.
1	0.71 (0.60, 0.83)	0.84 (0.63, 1.12)	1.24 (0.83, 1.65)	1.28 (0.68, 2.42)
2	0.81 (0.70, 0.91)	0.95 (0.73, 1.25)	1.35 (0.98, 1.73)	1.39 (0.76, 2.57)
3	0.76 (0.67, 0.86)	0.90 (0.69, 1.18)	1.48 (1.11, 1.84)	1.52 (0.83, 2.76)
4	0.83 (0.72, 0.94)	0.98 (0.75, 1.29)	0.90 (0.57, 1.23)	0.91 (0.47, 1.76)
5+	0.81 (0.67, 0.94)	0.95 (0.71, 1.27)	0.77 (0.35, 1.18)	0.78 (0.36, 1.68)
Education level				
High	0.70 (0.57, 0.82)	Ref.	1.18 (0.76, 1.60)	Ref.
Medium	0.77 (0.69, 0.85)	1.10 (0.90, 1.35)	1.33 (1.04, 1.62)	1.13 (0.74, 1.72)
Low	0.85 (0.78, 0.93)	1.22 (1.00, 1.49)	1.10 (0.86, 1.34)	0.93 (0.61, 1.41)
Cohabitation				
Living with partner	0.74 (0.67, 0.80)	Ref.	1.27 (1.04, 1.50)	Ref.
Living alone	0.85 (0.78, 0.92)	1.16 (1.02, 1.31)	1.10 (0.87, 1.34)	0.87 (0.66, 1.15)

CIP, cumulative incidence proportion; Ref., reference; SHR, subdistribution hazard ratio; VTE, venous thromboembolism.

### 3.3 Association between stroke severity and VTE

The results from all multivariable analyses of stroke severity for both stroke subtypes and both time periods are shown in Table 3. Overall, additional adjustment for age and sex had little to no effect on the association between stroke severity and VTE. Estimates did not meaningfully change in the multivariable model with predictors selected from the univariable analyses. This pattern was observed in patients with ischemic stroke and those with intracerebral hemorrhage for both phases.

**Table 3:** Subdistribution hazard ratios in multivariable analyses in acute and subacute phases after the index date for patients with ischemic stroke and intracerebral hemorrhage.

Stroke severity	Ischemic stroke (*N* = 129,345)			Intracerebral hemorrhage (*N* = 16,887)		
	Model 1: crude SHR (95% CI)	Model 2: adjusted SHR^a^ (95% CI)	Model 3: adjusted SHR (95% CI)	Model 1: crude SHR (95% CI)	Model 2: adjusted SHR^a^ (95% CI)	Model 3: adjusted SHR (95% CI)
Acute phase						
Mild	Ref.	Ref.	Ref.	Ref.	Ref.	Ref.
Moderate	3.28 (2.63, 4.09)	3.24 (2.57, 4.08)	3.03 (2.35, 3.92)^b^	4.07 (2.08, 7.98)	4.20 (2.17, 8.13)	4.19 (2.13, 8.24)^c^
Severe	4.48 (3.58, 5.61)	4.37 (3.41, 5.59)	4.83 (3.69, 6.33)^b^	2.41 (1.23, 4.74)	2.56 (1.29, 5.10)	2.51 (1.27, 4.99)^c^
Subacute phase						
Mild	Ref.	Ref.	Ref.	Ref.	Ref.	Ref.
Moderate	2.61 (2.25, 3.04)	2.66 (2.27, 3.11)	2.46 (2.07, 2.93)^d^	2.31 (1.61, 3.33)	2.34 (1.63, 3.37)	2.36 (1.64, 3.40)^c^
Severe	3.38 (2.89, 3.95)	3.44 (2.90, 4.09)	3.66 (3.04, 4.41)^d^	1.33 (0.92, 1.93)	1.37 (0.94, 2.00)	1.37 (0.94, 1.99)^c^

Ref., reference; SHR, subdistribution hazard ratio; VTE, venous thromboembolism.

a Adjusted for age and sex.

b Adjusted for selected predictors: stroke severity, age, sex, active cancer, previous VTE, smoking, recent trauma or surgery, postmenopausal hormone replacement therapy, education level, and cohabitation status.

c Adjusted for selected predictors: stroke severity, sex, active cancer, and previous VTE.

d Adjusted for selected predictors: stroke severity, age, sex, active cancer, previous VTE, smoking, recent trauma or surgery, and cohabitation status.

### 3.4 Sensitivity analyses

Supplementary Tables 6 and 7 show the results of the analyses restricted to patients with stroke who were not actively using anticoagulant therapy. The association between stroke severity and VTE was stronger in this study group compared with the original study group, for both ischemic stroke and intracerebral hemorrhage. The association between active cancer and VTE was also stronger, whereas the association between previous VTE and VTE was weaker. Similarly to findings in the main analyses, we did not observe substantial differences in SHRs for stroke severity from the univariable analyses after adjustment for both stroke subtypes and time periods (details in Supplemental Table 8).

## 4 Discussion

### 4.1 Principal findings

Our population-based cohort study indicated an increased risk of VTE ranging from 2-fold to 5-fold for stroke severity, active cancer, and previous VTE for both ischemic stroke and intracerebral hemorrhage, across both the subacute (3 months poststroke) and acute phases (7 days poststroke). The association between stroke severity and poststroke VTE remained strong after adjustment for both age and sex and predictors selected from univariable analyses.

### 4.2 Comparison with previous literature

Several studies have examined stroke severity and the risk of poststroke VTE in patients with ischemic stroke, but their findings on stroke severity as a predictor have been inconsistent [[14], [15], [16], [17], [18]]. Most of those studies were conducted in Asia, had short follow-up periods, and were at risk of selection bias (eg, by excluding patients who died within the first 7 days or were transferred to another hospital). A study by Kelly et al. [51], which used the Barthel Index to assess stroke severity, found that a subgroup of stroke patients with severe disability had an increased risk of VTE despite receiving standard thromboprophylaxis. Although the study had a small sample size, its findings are consistent with ours.

We found that patients with severe ischemic stroke were at highest risk of VTE, which is in line with several earlier studies [14,16,17]. In contrast, patients with moderate severity intracerebral hemorrhage were at highest risk of VTE compared with those with severe stroke. We are not aware of any other studies that evaluated stroke severity, as measured by a neurologic score scale, among patients with intracerebral hemorrhage. An explanation for the finding of moderate severity in patients with intracerebral hemorrhage having the highest risk might be a higher early mortality rate in the severe group than the moderate group. This may have resulted in a lower SHR for the severe group. Furthermore, our study showed that patients with intracerebral hemorrhage had a higher cumulative incidence of VTE compared with ischemic stroke patients. This may be explained by the differences in stroke severity; a greater proportion of intracerebral hemorrhage cases were classified as moderate to severe, whereas most ischemic stroke cases were mild. These findings support our conclusion that stroke severity is a key predictor of poststroke VTE—the more severe the stroke, the higher the likelihood of VTE.

Active cancer and previous VTE appeared to be strong predictors of VTE after stroke, both in the subacute and acute settings. These findings are consistent with those from existing studies in patients with ischemic stroke or stroke in general [1,4,13,18]. However, limited evidence is available regarding intracerebral hemorrhage alone. An American study of 2902 patients with intracerebral hemorrhage found a 6-fold elevated risk of developing VTE after stroke in patients who had a history of VTE. The study did not investigate cancer as a predictor [12].

### 4.3 Implications

The present study provides key information regarding clinical predictors of poststroke VTE, such as stroke severity, cancer, and previous VTE. These characteristics may aid clinicians in identifying patients at high risk of VTE after stroke. Notably, as stroke severity is assessed in every patient with stroke at admission, it is an important predictor applicable to all stroke patients.

Although VTE may be preventable with adequate thromboprophylaxis, continuing or initiating thromboprophylaxis after discharge is not yet common practice due to the inherent risk of bleeding. In subgroup analyses of randomized clinical trials, extended thromboprophylaxis for up to 4 weeks was associated with a 33% decreased risk of VTE among patients with ischemic stroke, corresponding to a number needed to treat of 40 [10]. Furthermore, the risk of major bleeding did not increase substantially by extending thromboprophylaxis from standard practice (major bleeding occurred in 1 more patient per 1000 in extended thromboprophylaxis compared to standard thromboprophylaxis) [10]. By identifying high-risk patients, our findings may help to improve the risk-benefit ratio of extended thromboprophylaxis beyond hospitalization. Additionally, future studies could build on these findings to develop a prediction model with relevant variables.

The benefit of extended thromboprophylaxis has been widely debated. For intracerebral hemorrhage, a survey from the United Kingdom has indicated that stroke physicians do not agree on the optimal method for VTE prevention. Some stroke physicians were uncertain about which patients should receive prophylaxis and the optimal type and duration of prophylaxis [37]. The correct answer should not necessarily be reduced to a single solution and is likely to vary by situation. Currently, guidelines do not advocate extended thromboprophylaxis, whereas for intracerebral hemorrhage, the decisions for each individual are almost completely at physicians’ discretion. Gaining a deeper understanding of clinical characteristics, particularly those predictive of adverse outcomes such as poststroke VTE, has the potential to assist clinicians in making better-informed decisions.

### 4.4 Limitations

This large population-based cohort study was based entirely on national registries with high quality and detailed data, and little to no loss of follow-up information. Nevertheless, our study should be interpreted within the context of its limitations. We had limited knowledge regarding medications during hospitalization and at other in-hospital facilities, such as hospital-based rehabilitation centers. Not all medications administered at hospitals is recorded in the registries; consequently, information on thromboprophylaxis during or after hospitalization was not available. The Danish Neurological Society’s guidelines are unclear for whom thromboprophylaxis is recommended during hospitalization, as well as the type and duration of thromboprophylaxis. Thus, our assumptions on thromboprophylaxis in hospitals were based on more general guidelines from the European Stroke Organisation or American Stroke Association [5,35,39]. These limitations might potentially have led to a misrepresentation of which patients actually received thromboprophylaxis during hospitalization, but it did not affect the main results of the study because we included all patients with stroke, regardless of their anticoagulant use. However, this aspect did limit our ability to further investigate the potential predictors in patients who did or did not receive thromboprophylaxis. Another limitation was the unavailable information on race/ethnicity, as ethnicity is not systematically recorded in the Patient Registry or Danish Civil Registration System [25,26]. The Danish population has a relatively stable and homogenous demography with regard to race.

Although the coding of first-time VTE diagnoses have high positive predictive value (88%) in the Patient Registry [52], distinguishing between first-time and recurrent VTE events is challenging. To decrease the risk of misclassification, we separated VTE events by time-related criteria in our study population (excluding patients with VTE event within the 90 days prior to the index date). Further, a relatively low sensitivity of VTE is expected in the Danish registries as only symptomatic events will receive medical attention and ultimately be coded. Due to this presumed underdiagnosis, the absolute risks in this study were almost certainly underestimated. For example, the CLOTS (The Clots in Legs Or sTockings after Stroke) trials showed a higher risk of poststroke VTE (∼10%) [53].

Immobilization is a well-established risk-factor for VTE. However, we were unable to identify valid surrogate markers for poststroke immobility [5]. We assume that diagnosis codes for paralysis primarily capture permanent deficits, whereas rather acute and potentially reversible impairments, more relevant in early poststroke phases, are not reliably recorded. It should also be noted that we did not to stratify VTE into deep vein thrombosis and pulmonary embolism in our analyses. Although such stratification could offer additional clinical insights, pulmonary embolism is notoriously difficult to diagnose. Consequently, undiagnosed fatal cases may not be captured in the registries, introducing considerably uncertainty into the estimates.

## 5 Conclusion

Our study shows that stroke severity, active cancer, and previous VTE are strong predictors of poststroke risk of VTE in both subacute and acute phases. Among these predictors, stroke severity is strongly associated with poststroke VTE even after adjustments. These findings offer valuable insights for clinicians and can be used to identify patients at elevated risk of VTE in whom extended thromboprophylaxis may be considered.

## References

[1] Corraini P., Ording A.G., Henderson V.W., Szépligeti S., Horváth-Puhó E., Sørensen H.T. (2016). Cancer, other comorbidity, and risk of venous thromboembolism after stroke: a population-based cohort study. Thromb Res.

[2] Rinde L.B., Småbrekke B., Mathiesen E.B., Løchen M.L., Njølstad I., Hald E.M. (2016). Ischemic stroke and risk of venous thromboembolism in the general population: the Tromsø Study. J Am Heart Assoc.

[3] Piazza G., Goldhaber S.Z., Kroll A., Goldberg R.J., Emery C., Spencer F.A. (2014). Venous thromboembolism in patients with prior stroke. Clin Appl Thromb Hemost.

[4] Pongmoragot J., Rabinstein A.A., Nilanont Y., Swartz R.H., Zhou L., Saposnik G. (2013). Investigators of Registry of Canadian Stroke Network (RCSN) and University of Toronto Stroke Program for Stroke Outcomes Research Canada (SORCan [www.sorcan.ca]) Working Group. Pulmonary embolism in ischemic stroke: clinical presentation, risk factors, and outcome. J Am Heart Assoc.

[5] Dennis M., Caso V., Kappelle L.J., Pavlovic A., Sandercock P., European Stroke Organisation (2016). European Stroke Organisation (ESO) guidelines for prophylaxis for venous thromboembolism in immobile patients with acute ischaemic stroke. Eur Stroke J.

[6] Cohen A.T., Harrington R.A., Goldhaber S.Z., Hull R.D., Wiens B.L., Gold A., APEX Investigators (2016). Extended thromboprophylaxis with betrixaban in acutely ill medical patients. N Engl J Med.

[7] Spyropoulos A.C., Ageno W., Albers G.W., Elliott C.G., Halperin J.L., Hiatt W.R., MARINER Investigators (2018). Rivaroxaban for thromboprophylaxis after hospitalization for medical illness. N Engl J Med.

[8] Cohen A.T., Spiro T.E., Büller H.R., Haskell L., Hu D., Hull R., MAGELLAN Investigators (2013). Rivaroxaban for thromboprophylaxis in acutely ill medical patients. N Engl J Med.

[9] Hull R.D., Schellong S.M., Tapson V.F., Monreal M., Samama M.M., Nicol P., EXCLAIM (Extended Prophylaxis for Venous ThromboEmbolism in Acutely Ill Medical Patients With Prolonged Immobilization) study (2010). Extended-duration venous thromboembolism prophylaxis in acutely ill medical patients with recently reduced mobility: a randomized trial. Ann Intern Med.

[10] Valeriani E., Potere N., Candeloro M., Spoto S., Porreca E., Rutjes A.W. (2022). Extended venous thromboprophylaxis in patients hospitalized for acute ischemic stroke: A systematic review and meta-analysis. Eur J Intern Med.

[11] Tøndel B.G., Morelli V.M., Hansen J.B., Braekkan S.K. (2022). Risk factors and predictors for venous thromboembolism in people with ischemic stroke: a systematic review. J Thromb Haemost.

[12] Ding D., Sekar P., Moomaw C.J., Comeau M.E., James M.L., Testai F. (2019). Venous thromboembolism in patients with spontaneous intracerebral hemorrhage: a multicenter study. Neurosurgery.

[13] Dennis M., Sandercock P., Reid J., Graham C., Murray G., Venables G., CLOTS Trials Collaboration (2011). Can clinical features distinguish between immobile patients with stroke at high and low risk of deep vein thrombosis? Statistical modelling based on the CLOTS trials cohorts. J Neurol Neurosurg Psychiatry.

[14] Balogun I.O., Roberts L.N., Patel R., Pathansali R., Kalra L., Arya R. (2016). Clinical and laboratory predictors of deep vein thrombosis after acute stroke. Thromb Res.

[15] Bembenek J., Karlinski M., Kobayashi A., Czlonkowska A. (2011). Early stroke-related deep venous thrombosis: risk factors and influence on outcome. J Thromb Thrombolysis.

[16] Ji R., Li G., Zhang R., Hou H., Zhao X., Wang Y. (2019). Higher risk of deep vein thrombosis after hemorrhagic stroke than after acute ischemic stroke. J Vasc Nurs.

[17] Wang Y., Shi Y., Dong Y., Dong Q., Ye T., Fang K. (2019). Clinical risk factors of asymptomatic deep venous thrombosis in patients with acute stroke. Clin Appl Thromb Hemost.

[18] Yin D., Shao P., Liu Y. (2016). Elevated lipoprotein (a) levels predict deep vein thrombosis in acute ischemic stroke patients. Neuroreport.

[19] Kammersgaard L.P. (2010). Survival after stroke. Risk factors and determinants in the Copenhagen Stroke Study. Dan Med Bull.

[20] Christensen H., Boysen G., Truelsen T. (2005). The Scandinavian stroke scale predicts outcome in patients with mild ischemic stroke. Cerebrovasc Dis.

[21] Skajaa N., Adelborg K., Horváth-Puhó E., Rothman K.J., Henderson V.W., Thygesen L.C. (2023). Labour market participation and retirement after stroke in Denmark: registry based cohort study. BMJ.

[22] Skajaa N., Adelborg K., Horváth-Puhó E., Rothman K.J., Henderson V.W., Thygesen L.C. (2022). Stroke and risk of mental disorders compared with matched general population and myocardial infarction comparators. Stroke.

[23] Schmidt M., Schmidt S.A.J., Adelborg K., Sundbøll J., Laugesen K., Ehrenstein V. (2019). The Danish health care system and epidemiological research: from health care contacts to database records. Clin Epidemiol.

[24] Johnsen S.P., Ingeman A., Hundborg H.H., Schaarup S.Z., Gyllenborg J. (2016). The Danish Stroke Registry. Clin Epidemiol.

[25] Schmidt M., Pedersen L., Sørensen H.T. (2014). The Danish Civil Registration System as a tool in epidemiology. Eur J Epidemiol.

[26] Schmidt M., Schmidt S.A.J., Sandegaard J.L., Ehrenstein V., Pedersen L., Sørensen H.T. (2015). The Danish National Patient Registry: a review of content, data quality, and research potential. Clin Epidemiol.

[27] Pottegård A., Schmidt S.A.J., Wallach-Kildemoes H., Sørensen H.T., Hallas J., Schmidt M. (2017). Data resource profile: the Danish National Prescription Registry. Int J Epidemiol.

[28] Jensen V.M., Rasmussen A.W. (2011). Danish Education Registers. Scand J Public Health.

[29] von Elm E., Altman D.G., Egger M., Pocock S.J., Gøtzsche P.C., Vandenbroucke J.P., STROBE Initiative (2008). The Strengthening the Reporting of Observational Studies in Epidemiology (STROBE) statement: guidelines for reporting observational studies. J Clin Epidemiol.

[30] Stroke Unit Trialists’ Collaboration (2007). Organised inpatient (stroke unit) care for stroke. Cochrane Database Syst Rev.

[31] Aguiar de Sousa D., von Martial R., Abilleira S., Gattringer T., Kobayashi A., Gallofré M. (2019). Access to and delivery of acute ischaemic stroke treatments: a survey of national scientific societies and stroke experts in 44 European countries. Eur Stroke J.

[32] Wildenschild C., Mehnert F., Thomsen R.W., Iversen H.K., Vestergaard K., Ingeman A. (2014). Registration of acute stroke: validity in the Danish Stroke Registry and the Danish National Registry of Patients. Clin Epidemiol.

[33] Hald S.M., Kring Sloth C., Hey S.M., Madsen C., Nguyen N., García Rodríguez L.A. (2018). Intracerebral hemorrhage: positive predictive value of diagnosis codes in two nationwide Danish registries. Clin Epidemiol.

[34] Damgaard D., Thomsen S.T. (2023). https://nnbv.dk/forebyggende-behandling-efter-iskaemisk-apopleksi-og-tci/.

[35] Kleindorfer D.O., Towfighi A., Chaturvedi S., Cockroft K.M., Gutierrez J., Lombardi-Hill D. (2021). 2021 Guideline for the prevention of stroke in patients with stroke and transient ischemic attack: a guideline from the American Heart Association/American Stroke Association. Stroke.

[36] Powers W.J., Rabinstein A.A., Ackerson T., Adeoye O.M., Bambakidis N.C., Becker K. (2019). Guidelines for the early management of patients with acute ischemic stroke: 2019 update to the 2018 guidelines for the early management of acute ischemic stroke: a guideline for healthcare professionals from the American Heart Association/American Stroke Association. Stroke.

[37] Mendel R., Abdelhameed N., Salman R.A.S., Cohen H., Dowlatshahi D., Freemantle N. (2023). Prevention of venous thromboembolism in acute spontaneous intracerebral haemorrhage: a survey of opinion. J Neurol Sci.

[38] Raslan A.M., Fields J.D., Bhardwaj A. (2010). Prophylaxis for venous thrombo-embolism in neurocritical care: a critical appraisal. Neurocrit Care.

[39] Greenberg S.M., Ziai W.C., Cordonnier C., Dowlatshahi D., Francis B., Goldstein J.N. (2022). American Heart Assoc./American Stroke Assoc. 2022 Guideline for the management of patients with spontaneous intracerebral hemorrhage: a guideline from the American Heart Association/American Stroke Association. Stroke.

[40] Steiner T., Al-Shahi Salman R., Beer R., Christensen H., Cordonnier C., Csiba L., European Stroke Organisation (2014). European Stroke Organisation (ESO) guidelines for the management of spontaneous intracerebral hemorrhage. Int J Stroke.

[41] Damgaard D. (2023). https://nnbv.dk/forebyggende-behandling-efter-ich/.

[42] Mulder F.I., Candeloro M., Kamphuisen P.W., Di Nisio M., Bossuyt P.M., Guman N. (2019). CAT-prediction collaborators. The Khorana score for prediction of venous thromboembolism in cancer patients: a systematic review and meta-analysis. Haematologica.

[43] Barbar S., Noventa F., Rossetto V., Ferrari A., Brandolin B., Perlati M. (2010). A risk assessment model for the identification of hospitalized medical patients at risk for venous thromboembolism: the Padua Prediction Score. J Thromb Haemost.

[44] CAPRIE Steering Committee (1996). A randomised, blinded, trial of clopidogrel versus aspirin in patients at risk of ischaemic events (CAPRIE). CAPRIE Steering Committee. Lancet.

[45] Scandinavian Stroke Study Group (1985). Multicenter trial of hemodilution in ischemic stroke--background and study protocol. Scandinavian stroke study group. Stroke.

[46] Govan L., Langhorne P., Weir C.J. (2009). Categorizing stroke prognosis using different stroke scales. Stroke.

[47] Severinsen M.T., Kristensen S.R., Overvad K., Dethlefsen C., Tjønneland A., Johnsen S.P. (2010). Venous thromboembolism discharge diagnoses in the Danish National Patient Registry should be used with caution. J Clin Epidemiol.

[48] Lau B., Cole S.R., Gange S.J. (2009). Competing risk regression models for epidemiologic data. Am J Epidemiol.

[49] Nicolaisen S.K., Thomsen R.W., Lau C.J., Sørensen H.T., Pedersen L. (2022). Development of a 5-year risk prediction model for type 2 diabetes in individuals with incident HbA1c-defined pre-diabetes in Denmark. BMJ Open Diabetes Res Care.

[50] Pedersen A.B., Mikkelsen E.M., Cronin-Fenton D., Kristensen N.R., Pham T.M., Pedersen L. (2017). Missing data and multiple imputation in clinical epidemiological research. Clin Epidemiol.

[51] Kelly J., Rudd A., Lewis R.R., Coshall C., Moody A., Hunt B.J. (2004). Venous thromboembolism after acute ischemic stroke: a prospective study using magnetic resonance direct thrombus imaging. Stroke.

[52] Sundbøll J., Adelborg K., Munch T., Frøslev T., Sørensen H.T., Bøtker H.E. (2016). Positive predictive value of cardiovascular diagnoses in the Danish National Patient Registry: a validation study. BMJ Open.

[53] Dennis M., Sandercock P.A., Reid J., Graham C., Murray G., CLOTS Trials Collaboration (2009). Effectiveness of thigh-length graduated compression stockings to reduce the risk of deep vein thrombosis after stroke (CLOTS trial 1): a multicentre, randomised controlled trial. Lancet.

