# Supplementary material for: Stroke severity and other predictors of venous thromboembolism in stroke patients—a population-based cohort study
Source: Res Pract Thromb Haemost. 2025 Oct 10;9(7):103220. doi: 10.1016/j.rpth.2025.103220 (PMC12634850; doi:10.1016/j.rpth.2025.103220)
Supplement: Supplementary Material [file mmc1.docx]

**SUPPLEMENTARY MATERIAL**

Hansen, DV; van Es, N; Sørensen, HT; Coutinho JM; Skajaa, N.

**Stroke severity and other predictors of venous thromboembolism in stroke patients - a population-based cohort study**

**Table of Contents**

[Supplementary Table 1. Danish registries 2](#_Toc159407308)

[Supplementary Table 2. Codes and definitions used in this study. 3](#_Toc159407309)

[Supplementary Table 3. Description of Essen Risk Score. 5](#_Toc159407310)

[Supplementary Table 4. Scandinavian Stroke Scale. 6](#_Toc159407311)

[Supplementary Table 5. Risks and subdistribution hazards ratios for venous thromboembolism in the acute phase for ischemic stroke and intracerebral hemorrhage. 8](#_Toc159407312)

[Supplementary Table 6. Sensitivity analyses for a restricted study population not actively using anticoagulant therapy. Risks and subdistribution hazard ratios for venous thromboembolism in the acute phase for ischemic stroke and intracerebral hemorrhage. 9](#_Toc159407313)

[Supplementary Table 7. Sensitivity analyses for a restricted study population not actively using anticoagulant therapy. Risks and subdistribution hazard ratios for venous thromboembolism in the subacute phase for ischemic stroke and intracerebral hemorrhage. 10](#_Toc159407314)

[Supplementary Table 8. Sensitivity analyses with a study population not actively using anticoagulant therapy. Subdistribution hazard ratios in multivariable analyses at the acute and subacute phase for ischemic stroke and intracerebral hemorrhage. 11](#_Toc159407315)

**Supplementary Table 1.** Danish registries

**The Danish Stroke Registry:** The Danish Stroke Registry is a nationwide clinical quality database established in 2003, with complete data from May 2004. Reporting to this registry is mandatory for all Danish hospitals treating patients with acute stroke, as defined by the World Health Organization criteria. Approximately 90% of all strokes in Denmark are captured by this registry.

**The Danish Civil Registration System:** This registry contains data on demographic information, vital status, and migration from the entire Danish Population.

**The Danish National Patient Registry:** This registry contains both administrative and clinical data with complete, nationwide information on hospital inpatients since 1977, as well as outpatient clinic and emergency contacts since 1995. Diagnoses are recorded once as either primary or secondary and are classified according to the International Classification of Disease, Eighth Revision (ICD-8) through 1993, and Tenth Revision (ICD-10) thereafter.

**The Danish National Prescription Registry:** This registry contains all prescriptions redeemed by Danish residents at community pharmacies since 1995. The data include information on drug type according to the Anatomical Therapeutic Chemical (ATC) code as well as the dispensing date of the redeemed prescription.

**The Attainment Register:** This registry is annual and contains data on the highest completed level of education since 1981.

**Supplementary Table 2.** Codes and definitions used in this study.

|  | **Code or variable** | **Definition** | **Registry used** |
| --- | --- | --- | --- |
| **Study population** |  |  |  |
| Ischemic stroke | Apotype: 3, (ICD-10 equivalent codes: I63) | Record indicating hospitalization with acute stroke | DSR |
| Intracerebral hemorrhage | Apotype: 2 (ICD-10 equivalent codes: I61) | Record indicating hospitalization with acute stroke | DSR |
| **Exclusion criteria** |  |  |  |
| Venous thromboembolism | ICD-10 codes: I801-I803, I26  ICD-8 codes: 45100, 45099 | Diagnosis (any type) 90 days before the index date | DNPR |
| Stroke | Apotype: 2, 3, 4  ICD-10 codes: I60, I61, I63, I64  ICD-8 codes: 430, 431, 433, 434) | Record indicating hospitalization with acute stroke OR diagnosis (any type) before the index date | DSR, DNPR |
| **Potential predictors** |  |  |  |
| Stroke severity | INDTOTAL | Stroke severity, assessed with the Scandinavian Stroke Scale at hospital admission, divided into three severity levels (mild, moderate, and severe) | DSR |
| Age | Date of stroke diagnosis, date of birth | Age at date of stroke diagnosis | DSR, CRS |
| Sex | KOEN | Male or female | CRS |
| Active cancer | ICD-10 codes: C00-C43, C45-C99, D45, D473 | Cancer diagnosis (except non-melanoma skin cancer) or metastasis within 180 days before the index date; includes polycythemia vera and essential thrombocythemia. | DNPR |
| Previous venous thromboembolism | ICD-10 codes: I801-I803, I26 | Diagnosis (any type) before the index date, except for 90 days before the index date |  |
| Smoking | RYGNING | Current/occasional, former, never (patients’ smoking status prior to hospitalization) | DSR |
| Recent trauma/surgery | ICD-10 codes: S00-T14  NOMESCO classification after 1996: KA-KQ, KX, KY | Diagnosis with trauma, fracture, or surgery within 8 weeks before the index date | DNPR |
| Heart failure | ICD-10 codes: I500-I503, I508, I509, I110, I130, I132, I420, I426-I429 | Diagnosis (any type) before the index date | DNPR |
| Chronic kidney disease | ICD-10 codes: N03, N11, N18-N19 | Diagnosis (any type) before the index date | DNPR |
| Obstructive pulmonary disease | ICD-10 codes: J40-J46  ATC codes: R03 | Diagnosis (any type) before the index date OR two or more prescriptions in the year before the index date | DNPR, NPR |
| Chronic inflammatory disease | ICD-10 codes: M05-M06, M08-M09, M30-M36, D86, K50-K51 | Diagnosis (any type) before the index date | DNPR |
| Paralysis | ICD-10 codes: G81-G82 | Diagnosis (any type) before the index date | DNPR |
| Hormone replacement therapy | ATC codes: G02BA03, G03CA03, G03CA04, G03CA53, G03CA57, G03CB01, G03DA02, G03DA04, G03DC02, G03DC03, G03DC05, G03FA01, G03FA12; G03FB01, G03FB05, G03FB06, G03FB09, G03HB01, G03XC01 | One or more prescriptions in the 3 months before the index date | NPR |
| ***Essen risk score*** |  |  |  |
| Hypertension | ICD-10 codes: I10-I13, I15  ATC codes: C02-C04, C07-C09 | Diagnosis (any type) before the index date OR two or more prescriptions in the year before the index date | DNPR, NPR |
| Diabetes mellitus | ICD-10 codes: E10-E14  ATC codes: A10A, A10B | Diagnosis (any type) before the index date OR two or more prescriptions in the year before the index date | DNPR, NPR |
| Myocardial infarction | ICD-10 codes: I21-I23 | Diagnosis (any type) before the index date | DNPR |
| Other CVD | ICD-10 codes: I20, I24-I25, I48, I50, E78  ATC codes: C10, C01DA | Diagnosis (any type) before the index date OR two or more prescriptions in the year before the index date | DNPR, NPR |
| Peripheral artery disease | ICD-10 codes: I70-I74 | Diagnosis (any type) before the index date | DNPR |
| Current smoking | RYGNING | Current/occasional, or former/never | DSR |
| ***Demographics*** |  |  |  |
| Cohabitation status | FAMILIE_TYPE | Cohabitation status in the year before the index year (living alone or living with partner) | CRS |
| Highest achieved education | HFAUDD | Education groups (high, medium, low), calculated according to DISCED levels; the most recent data were used at least 1 year before the index date | UDDA |
| **Outcomes** |  |  |  |
| Venous thromboembolism | ICD-10 codes: I801-I803, I26 | Diagnosis (inpatient/outpatient, primary/secondary) in the follow up time after or on the index date | DNPR |
| Deep-vein thrombosis | ICD-10 codes: I801-I803 | Diagnosis (inpatient/outpatient, primary/secondary) in the follow up time after or on the index date | DNPR |
| Pulmonary embolism | ICD-10 codes: I26 | Diagnosis (inpatient/outpatient, primary/secondary) in the follow up time after or on the index date | DNPR |
| **Sensitivity analyses** |  |  |  |
| Atrial fibrillation/flutter or active use of anticoagulant therapy | ICD-10 codes: I48  ATC codes: B01AA, B01AB, B01AE07, B01AF | Diagnosis (any type) before index date OR one or more prescriptions redeemed in the 90 days before the index date; one or more prescriptions redeemed after the index date (censoring) |  |
|  |  |  |  |

**Abbreviations**: ICD: International Classification of Disease; ATC: Anatomical Therapeutic Chemical; DSR: Danish Stroke Registry; DNPR: Danish National Patient Registry; CRS: Civil Registration System; UDDA: the Attainment Register; NPR: Danish National Prescription Registry.

**Supplementary Table 3.** Description of Essen Risk Score.

| Variables in the Essen Score | Points |
| --- | --- |
| Age, y |  |
| < 65 | 0 |
| 65–75 | 1 |
| > 75 | 2 |
| History of hypertension | 1 |
| History of diabetes mellitus | 1 |
| History of peripheral artery disease | 1 |
| History of previous myocardial infarction | 1 |
| History of other cardiovascular disease (except myocardial infarction) | 1 |
| Current smoking | 1 |
| Additional TIA or ischemic stroke in addition to qualifying event (i.e., previous TIA/ischemic stroke **(excluded in our study**) | (1) |

**Abbreviation:** TIA: transient ischemic attack

**Supplementary Table 4.** Scandinavian Stroke Scale.

| **Functions** | **Scores** |
| --- | --- |
| **Consciousness** |  |
| Fully conscious | 6 |
| Somnolent, can be awakened to full consciousness | 4 |
| Reacts to verbal commands but is not fully conscious | 2 |
|  |  |
| **Eye movement** |  |
| No gaze palsy | 4 |
| Gaze palsy present | 2 |
| Conjugate eye deviation | 0 |
|  |  |
| **Arm, motor power*** |  |
| Raises arm with normal strength | 6 |
| Raises arm with reduced strength | 5 |
| Raises arm with elbow flexion | 4 |
| Can move, but not against gravity | 2 |
| Paralysis | 0 |
|  |  |
| **Hand, motor power*** |  |
| Normal strength | 6 |
| Reduced strength in full range | 4 |
| Some movement, fingertips do not reach palm | 2 |
| Paralysis | 0 |
|  |  |
| **Leg, motor power*** |  |
| Normal strength | 6 |
| Raises straight leg with reduced strength | 5 |
| Raises leg with flexion of knee | 4 |
| Can move, but not against gravity | 2 |
| Paralysis  **Orientation** | 0 |
| Correct for time, place, and person | 6 |
| Two of these | 4 |
| One of these | 2 |
| Completely disoriented | 0 |
|  |  |
| **Speech** |  |
| No aphasia | 10 |
| Limited vocabulary or incoherent speech | 6 |
| More than yes/no, but no longer sentences | 3 |
| Only yes/no or less | 0 |
|  |  |
| **Facial palsy** |  |
| None/dubious | 2 |
| Present | 0 |
|  |  |
| **Gait** |  |
| Walks 5 min without aids | 12 |
| Walks with aids | 9 |
| Walks with help of another person | 6 |
| Sits without support | 3 |
| Bedridden/wheelchair | 0 |
|  |  |
| **Total score**  *****Motor power is assessed on only the affected side |  |

**Supplementary Table 5.** Risks and subdistribution hazards ratios for venous thromboembolism in the acute phase for ischemic stroke and intracerebral hemorrhage.

|  | **Ischemic stroke (N = 129,345)** | | **Intracerebral hemorrhage (N = 16,887)** | |
| --- | --- | --- | --- | --- |
| **Potential predictors** | Risk, % (95% CI) | Crude SHR (95% CI) | Risk, % (95% CI) | Crude SHR (95% CI) |
| Stroke severity |  |  |  |  |
| Mild | 0.18 (0.15, 0.21) | Ref. | 0.14 (0.04, 0.24) | Ref. |
| Moderate | 0.67 (0.55, 0.79) | 3.28 (2.63, 4.09) | 0.73 (0.42, 1.05) | 4.07 (2.08, 7.98) |
| Severe | 0.99 (0.80, 1.17) | 4.48 (3.58, 5.61) | 0.54 (0.33, 0.76) | 2.41 (1.23, 4.74) |
| Age |  |  |  |  |
| < 65 years | 0.24 (0.19, 0.29) | Ref. | 0.46 (0.26, 0.66) | Ref. |
| 65–79 years | 0.38 (0.32, 0.44) | 1.59 (1.25, 2.03) | 0.41 (0.24, 0.58) | 0.80 (0.47, 1.36) |
| ≥ 80 years | 0.45 (0.38, 0.53) | 1.91 (1.49, 2.44) | 0.44 (0.23, 0.66) | 0.78 (0.45, 1.37) |
| Sex |  |  |  |  |
| Female | 0.42 (0.36, 0.47) | Ref. | 0.27 (0.15, 0.39) | Ref. |
| Male | 0.30 (0.25, 0.34) | 0.71 (0.59, 0.85) | 0.59 (0.41, 0.77) | 1.66 (1.05, 2.64) |
| Active cancer |  |  |  |  |
| No | 0.33 (0.29, 0.36) | Ref. | 0.41 (0.30, 0.52) | Ref. |
| Yes | 1.04 (0.71, 1.36) | 2.90 (2.15, 3.90) | 1.22 (0.16, 2.29) | 2.72 (1.25, 5.92) |
| Previous VTE |  |  |  |  |
| No | 0.34 (0.30, 0.37) | Ref. | 0.39 (0.28, 0.49) | Ref. |
| Yes | 0.93 (0.60, 1.26) | 3.73 (2.85, 4.87) | 2.54 (0.80, 4.28) | 5.51 (3.09, 9.83) |
| Smoking |  |  |  |  |
| Never | 0.39 (0.32, 0.45) | Ref. | 0.44 (0.24, 0.64) | Ref. |
| Current/occasional | 0.23 (0.18, 0.28) | 0.59 (0.46, 0.75) | 0.38 (0.16, 0.61) | 0.78 (0.40, 1.52) |
| Former | 0.42 (0.34, 0.50) | 1.05 (0.84, 1.31) | 0.53 (0.26, 0.79) | 1.14 (0.64, 2.01) |
| Recent trauma, fracture, or surgery (8 weeks before) |  |  |  |  |
| No | 0.34 (0.30, 0.37) | Ref. | 0.42 (0.30, 0.53) | Ref. |
| Yes | 0.52 (0.37, 0.68) | 1.65 (1.26, 2.14) | 0.66 (0.17, 1.16) | 1.43 (0.71, 2.87) |
| Heart failure |  |  |  |  |
| No | 0.35 (0.31, 0.38) | Ref. | 0.44 (0.33, 0.55) | Ref. |
| Yes | 0.51 (0.30, 0.72) | 1.29 (0.93, 1.78) | 0.3 (0.00, 0.89) | 0.48 (0.12, 1.96) |
| Chronic kidney disease |  |  |  |  |
| No | 0.35 (0.32, 0.39) | Ref. | 0.44 (0.33, 0.56) | Ref. |
| Yes | 0.35 (0.11, 0.59) | 0.85 (0.47, 1.54) | 0.00 (0.00, 0.00) | 0.95 (0.23, 3.86) |
| Chronic obstructive pulmonary disease |  |  |  |  |
| No | 0.34 (0.30, 0.38) | Ref. | 0.42 (0.31, 0.54) | Ref. |
| Yes | 0.42 (0.32, 0.53) | 1.24 (0.98, 1.57) | 0.52 (0.18, 0.86) | 1.03 (0.54, 1.95) |
| Chronic inflammatory disease |  |  |  |  |
| No | 0.36 (0.32, 0.39) | Ref. | 0.42 (0.31, 0.53) | Ref. |
| Yes | 0.28 (0.14, 0.41) | 0.79 (0.52, 1.22) | 0.78 (0.10, 1.45) | 1.81 (0.83, 3.93) |
| Paralysis |  |  |  |  |
| No | 0.35 (0.32, 0.39) | Ref. | 0.43 (0.32, 0.54) | Ref. |
| Yes | 0.37 (0.00, 1.10) | 0.85 (0.12, 6.04) | 2.63 (0.00, 7.72) | 4.75 (0.66, 34.44) |
| Postmenopausal hormone replacement therapy |  |  |  |  |
| No | 0.34 (0.31, 0.38) | Ref. | 0.45 (0.33, 0.56) | Ref. |
| Yes | 0.57 (0.36, 0.77) | 1.53 (1.08, 2.18) | 0.15 (0.00, 0.45) | 0.27 (0.04, 1.92) |
| Essen risk score |  |  |  |  |
| 0 | 0.26 (0.15, 0.36) | Ref. | 0.38 (0.05, 0.70) | Ref. |
| 1 | 0.31 (0.23, 0.39) | 1.18 (0.72, 1.91) | 0.50 (0.24, 0.77) | 1.02 (0.42, 2.50) |
| 2 | 0.35 (0.28, 0.42) | 1.37 (0.86, 2.18) | 0.41 (0.20, 0.63) | 0.84 (0.35, 2.05) |
| 3 | 0.39 (0.32, 0.46) | 1.55 (0.98, 2.43) | 0.58 (0.33, 0.83) | 1.30 (0.57, 2.98) |
| 4 | 0.36 (0.27, 0.45) | 1.58 (1.00, 2.51) | 0.21 (0.00, 0.42) | 0.36 (0.12, 1.06) |
| 5+ | 0.40 (0.28, 0.52) | 1.52 (0.94, 2.46) | 0.31 (0.00, 0.67) | 0.55 (0.17, 1.72) |
| Educational level |  |  |  |  |
| High | 0.27 (0.19, 0.35) | Ref. | 0.29 (0.06, 0.52) | Ref. |
| Medium | 0.32 (0.27, 0.37) | 1.10 (0.80, 1.50) | 0.54 (0.34, 0.74) | 1.77 (0.82, 3.83) |
| Low | 0.41 (0.36, 0.47) | 1.47 (1.09, 1.98) | 0.40 (0.24, 0.57) | 1.39 (0.64, 3.02) |
| Cohabitation |  |  |  |  |
| Living with partner | 0.31 (0.27, 0.36) | Ref. | 0.46 (0.30, 0.61) | Ref. |
| Living alone | 0.40 (0.35, 0.46) | 1.36 (1.14, 1.63) | 0.41 (0.26, 0.57) | 0.82 (0.52, 1.28) |

**Supplementary Table 6.** Sensitivity analyses for a restricted study population not actively using anticoagulant therapy. Risks and subdistribution hazard ratios for venous thromboembolism in the acute phase for ischemic stroke and intracerebral hemorrhage.

|  | **Ischemic stroke (N = 111,092)** | | **Intracerebral hemorrhage (N = 138,10)** | |
| --- | --- | --- | --- | --- |
| **Potential predictors** | CIP, % (95% CI) | Crude SHR (95% CI) | CIP, % (95% CI) | Crude SHR (95% CI) |
| Stroke severity |  |  |  |  |
| Mild | 0.18 (0.15, 0.21) | Ref. | 0.14 (0.04, 0.24) | Ref. |
| Moderate | 0.67 (0.55, 0.79) | 3.68 (2.88, 4.71 | 0.73 (0.42, 1.05) | 5.21 (2.21, 12.26) |
| Severe | 0.99 (0.80, 1.17) | 5.32 (4.14, 6.85) | 0.54 (0.33, 0.76) | 3.57 (1.53, 8.30) |
| Age |  |  |  |  |
| < 65 years | 0.24 (0.19, 0.29) | Ref. | 0.46 (0.26, 0.66) | Ref. |
| 65–79 years | 0.38 (0.32, 0.44) | 1.58 (1.22, 2.05) | 0.41 (0.24, 0.58) | 0.88 (0.48, 1.60) |
| ≥ 80 years | 0.45 (0.38, 0.53) | 1.90 (1.44, 2.49) | 0.44 (0.23, 0.66) | 0.95 (0.50, 1.79) |
| Sex |  |  |  |  |
| Female | 0.42 (0.36, 0.47) | Ref. | 0.27 (0.15, 0.39) | Ref. |
| Male | 0.30 (0.25, 0.34) | 0.72 (0.59, 0.87) | 0.59 (0.41, 0.77) | 2.22 (1.28, 3.85) |
| Active cancer |  |  |  |  |
| No | 0.33 (0.29, 0.36) | Ref. | 0.41 (0.30, 0.52) | Ref. |
| Yes | 1.04 (0.71, 1.36) | 3.15 (2.25, 4.40) | 1.22 (0.16, 2.29) | 2.95 (1.18, 7.38) |
| Previous VTE |  |  |  |  |
| No | 0.34 (0.30, 0.37) | Ref. | 0.39 (0.28, 0.49) | Ref. |
| Yes | 0.93 (0.60, 1.26) | 2.78 (1.92, 4.03) | 2.54 (0.80, 4.28) | 6.68 (3.17, 14.06) |
| Smoking |  |  |  |  |
| Never | 0.39 (0.32, 0.45) | Ref. | 0.44 (0.24, 0.64) | Ref. |
| Current/occasional | 0.23 (0.18, 0.28) | 0.60 (0.46, 0.79) | 0.38 (0.16, 0.61) | 0.85 (0.40, 1.79) |
| Former | 0.42 (0.34, 0.50) | 1.08 (0.84, 1.39) | 0.53 (0.26, 0.79) | 1.19 (0.60, 2.34) |
| Recent trauma, fracture, or surgery (8 weeks before) |  |  |  |  |
| No | 0.34 (0.30, 0.72) | Ref. | 0.42 (0.30, 0.53) | Ref. |
| Yes | 0.52 (0.37, 0.68) | 1.54 (1.12, 2.11) | 0.66 (0.17, 1.16) | 1.61 (0.73, 3.54) |
| Heart failure |  |  |  |  |
| No | 0.35 (0.31, 0.38) | Ref. | 0.44 (0.33, 0.55) | Ref. |
| Yes | 0.51 (0.30, 0.72) | 1.47 (0.96, 2.26) | 0.30 (0.00, 0.89) | 0.67 (0.09, 4.82) |
| Chronic kidney disease |  |  |  |  |
| No | 0.35 (0.32, 0.39) | Ref. | 0.44 (0.33, 0.56) | Ref. |
| Yes | 0.35 (0.11, 0.59) | 0.98 (0.49, 1.98) | 0.00 (0.00, 0.00) | - |
| Chronic obstructive pulmonary disease |  |  |  |  |
| No | 0.34 (0.30, 0.38) | Ref. | 0.42 (0.31, 0.54) | Ref. |
| Yes | 0.42 (0.32, 0.53) | 1.24 (0.95, 1.63) | 0.52 (0.18, 0.86) | 1.21 (0.60, 2.46) |
| Chronic inflammatory disease |  |  |  |  |
| No | 0.36 (0.32, 0.39) | Ref. | 0.42 (0.31, 0.53) | Ref. |
| Yes | 0.28 (0.14, 0.41) | 0.77 (0.47, 1.27) | 0.78 (0.10, 1.45) | 1.85 (0.74, 4.62) |
| Paralysis |  |  |  |  |
| No | 0.35 (0.32, 0.39) | Ref. | 0.43 (0.32, 0.54) | Ref. |
| Yes | 0.37 (0.00, 1.10) | 1.07 (0.15, 7.61) | 2.63 (0.00, 7.72) | 6.02 (0.83, 43.81) |
| Postmenopausal hormone replacement therapy |  |  |  |  |
| No | 0.34 (0.31, 0.38) | Ref. | 0.45 (0.33, 0.56) | Ref. |
| Yes | 0.57 (0.36, 0.77) | 1.65 (1.12, 2.43) | 0.15 (0.00, 0.45) | 0.34 (0.05, 2.46) |
| Essen risk score |  |  |  |  |
| 0 | 0.26 (0.15, 0.36) | Ref. | 0.38 (0.05, 0.70) | Ref. |
| 1 | 0.31 (0.23, 0.39) | 1.21 (0.74,1.98) | 0.50 (0.24, 0.77) | 1.35 (0.49, 3.75) |
| 2 | 0.35 (0.28, 0.42) | 1.38 (0.86, 2.22) | 0.41 (0.20, 0.63) | 1.10 (0.39, 3.05) |
| 3 | 0.39 (0.32, 0.46) | 1.51 (0.95, 2.42) | 0.58 (0.33, 0.83) | 1.53 (0.57, 4.08) |
| 4 | 0.36 (0.27, 0.45) | 1.41 (0.86, 2.32) | 0.21 (0.00, 0.42) | 0.55 (0.15, 2.05) |
| 5+ | 0.40 (0.28, 0.52) | 1.56 (0.93, 2.62) | 0.31 (0.00, 0.67) | 0.82 (0.20, 3.43) |
| Educational level |  |  |  |  |
| High | 0.27 (0.19, 0.35) | Ref. | 0.29 (0.06, 0.52) | Ref. |
| Medium | 0.32 (0.27, 0.37) | 1.19 (0.83, 1.69) | 0.54 (0.34, 0,74) | 1.87 (0.77, 4.52) |
| Low | 0.41 (0.36, 0.47) | 1.54 (1.10, 2.17) | 0.40 (0.24, 0.57) | 1.38 (0.56, 3.40) |
| Cohabitation |  |  |  |  |
| Living with partner | 0.31 (0.27, 0.36) | Ref. | 0.46 (0.30, 0.61) | Ref. |
| Living alone | 0.40 (0.35, 0.46) | 1.29 (1.05, 1.57) | 0.41 (0.26, 0.57) | 0.91 (0.54, 1.51) |

**Supplementary Table 7.** Sensitivity analyses for a restricted study population not actively using anticoagulant therapy. Risks and subdistribution hazard ratios for venous thromboembolism in the subacute phase for ischemic stroke and intracerebral hemorrhage.

|  | **Ischemic stroke (N = 111,092)** | | **Intracerebral hemorrhage (N = 13,810)** | |
| --- | --- | --- | --- | --- |
| **Potential predictors** | CIP, % (95% CI) | Crude SHR (95% CI) | CIP, % (95% CI) | Crude SHR (95% CI) |
| Stroke severity |  |  |  |  |
| Mild | 0.45 (0.40, 0.50) | Ref. | 0.61 (0.39, 0.82) | Ref. |
| Moderate | 1.34 (1.16, 1.51) | 3.02 (2.55, 3.58) | 1.84 (1.34, 2.33) | 3.04 (1.94, 4.77) |
| Severe | 1.90 (1.64, 2.17) | 4.22 (3.54, 5.04) | 1.17 (0.85, 1.49) | 1.87 (1.19, 2.93) |
| Age |  |  |  |  |
| < 65 years | 0.60 (0.52, 0.68) | Ref. | 0.92 (0.65, 1.20) | Ref. |
| 65–79 years | 0.79 (0.71, 0.87) | 1.33 (1.12, 1.58) | 1.24 (0.94, 1.53) | 1.33 (0.90, 1.96) |
| ≥ 80 years | 0.90 (0.78, 1.01) | 1.51 (1.25, 1.81) | 1.03 (0.71, 1.36) | 1.10 (0.71, 1.71) |
| Sex |  |  |  |  |
| Female | 0.86 (0.77, 0.94) | Ref. | 0.86 (0.64, 1.09) | Ref. |
| Male | 0.67 (0.60, 0.73) | 0.78 (0.68, 0.90) | 1.28 (1.01, 1.54) | 1.49 (1.08, 2.08) |
| Active cancer |  |  |  |  |
| No | 0.70 (0.65, 0.76) | Ref. | 1.03 (0.85, 1.20) | Ref. |
| Yes | 2.16 (1.68, 2.65) | 3.09 (2.44, 3.91) | 2.72 (1.13, 4.31) | 2.66 (1.44, 4.92) |
| Previous VTE |  |  |  |  |
| No | 0.70 (0.65, 0.75) | Ref. | 0.98 (0.81, 1.15) | Ref. |
| Yes | 2.46 (1.91, 3.01) | 3.51 (2.77, 4.45) | 5.19 (2.71, 7.66) | 5.44 (3.24, 9.16) |
| Smoking |  |  |  |  |
| Never | 0.83 (0.73, 0.93) | Ref. | 1.11 (0.79, 1.42) | Ref. |
| Current/occasional | 0.51 (0.44, 0.59) | 0.62 (0.51, 0.74) | 0.80 (0.48, 1.30) | 0.72 (0.44, 1.19) |
| Former | 0.89 (0.78, 1.01) | 1.07 (0.90, 1.28) | 1.10 (0.72, 1.49) | 0.99 (0.63, 1.56) |
| Recent trauma, fracture, or surgery (8 weeks before) |  |  |  |  |
| No | 0.72 (0.67, 0.78) | Ref. | 1.08 (0.90, 1.26) | Ref. |
| Yes | 1.12 (0.89, 1.35) | 1.55 (1.25, 1.94) | 1.05 (0.43, 1.67) | 0.99 (0.53, 1.82) |
| Heart failure |  |  |  |  |
| No | 0.74 (0.69, 0.80) | Ref. | 1.09 (0.91, 1.26) | Ref. |
| Yes | 1.00 (0.70, 1.31) | 1.35 (0.99, 1.85) | 0.61 (0.00, 1.45) | 0.56 (0.14, 2.26) |
| Chronic kidney disease |  |  |  |  |
| No | 0.75 (0.70, 0.80) | Ref. | 1.09 (0.92, 1.27) | Ref. |
| Yes | 0.77 (0.40, 1.13) | 1.02 (0.63, 1.65) | 0.35 (0.00, 1.07) | 0.33 (0.05, 2.34) |
| Chronic obstructive pulmonary disease |  |  |  |  |
| No | 0.74 (0.68, 0.79) | Ref. | 1.09 (0.90, 1.27) | Ref. |
| Yes | 0.85 (0.70, 1.01) | 1.14 (0.94, 1.39) | 1.10 (0.29, 1.90) | 0.91 (0.55, 1.51) |
| Chronic inflammatory disease |  |  |  |  |
| No | 0.74 (0.69, 0.80) | Ref. | 1.08 (0.90, 1.25) | Ref. |
| Yes | 0.90 (0.65, 1.15) | 1.20 (0.90, 1.60) | 1.10 (0.29, 1.90) | 1.02 (0.48, 2.19) |
| Paralysis |  |  |  |  |
| No | 0.75 (0.70, 0.81) | Ref. | 1.06 (0.89, 1.24) | Ref. |
| Yes | 0.37 (0.00, 1.10) | 0.52 (0.07, 3.70) | 5.26 (0.00, 12.36) | 5.01 (1.24, 20.25) |
| Postmenopausal hormone replacement therapy |  |  |  |  |
| No | 0.74 (0.69, 0.79) | Ref. | 1.10 (0.92, 1.28) | Ref. |
| Yes | 1.00 (0.72, 1.28) | 1.36 (1.01, 1.82) | 0.62 (0.01, 1.23) | 0.56 (0.21, 1.52) |
| Essen risk score |  |  |  |  |
| 0 | 0.77 (0.57, 0.96) | Ref. | 0.83 (0.34, 1.33) | Ref. |
| 1 | 0.69 (0.57, 0.81) | 0.91 (0.67, 1.23) | 1.16 (0.76, 1.56) | 1.40 (0.70, 2.77) |
| 2 | 0.78 (0.67, 0.89) | 1.02 (0.77, 1.36) | 122 (0.85, 1.59) | 1.46 (0.75, 2.84) |
| 3 | 0.72 (0.62, 0.82) | 0.95 (0.71, 1.26) | 1.26 (0.88, 1.63) | 1.51 (0.78, 2.92) |
| 4 | 0.81 (0.67, 0.95) | 1.06 (0.78, 1.44) | 0.70 (0.32, 1.08) | 0.82 (0.37, 1.84) |
| 5+ | 0.78 (0.62, 0.95) | 1.03 (0.40, 1.43) | 0.74 (0.19, 1.29) | 0.88 (0.34, 2.27) |
| Educational level |  |  |  |  |
| High | 0.63 (0.50, 0.76) | Ref. | 0.97 (0.55, 1.39) | Ref. |
| Medium | 0.75 (0.67, 0.83) | 1.19 (0.94, 1.50) | 1.25 (0.94, 1.56) | 1.29 (0.78, 2.14) |
| Low | 0.81 (0.73, 0.89) | 1.29 (1.02, 1.62) | 1.01 (0.75, 1.27) | 1.04 (0.62, 1.73) |
| Cohabitation |  |  |  |  |
| Living with partner | 0.72 (0.65, 0.79) | Ref. | 1.12 (0.88, 1.36) | Ref. |
| Living alone | 0.79 (0.71, 0.87) | 1.10 (0.96, 1.26) | 1.03 (0.78, 1.28) | 0.92 (0.66, 1.27) |

**Supplementary Table 8.** Sensitivity analyses with a study population not actively using anticoagulant therapy. Subdistribution hazard ratios in multivariable analyses at the acute and subacute phase for ischemic stroke and intracerebral hemorrhage.

|  | **Ischemic stroke (N = 111,092)** | | | **Intracerebral hemorrhage (N = 13,810)** | | |
| --- | --- | --- | --- | --- | --- | --- |
| **Stroke severity** | Model 1:  crude SHR (95% CI) | Model 2: adjusted SHR  (95% CI) ^a^ | Model 3:  adjusted SHR  (95% CI) | Model 1:  crude SHR (95% CI) | Model 2:  adjusted SHR (95% CI) ^a^ | Model 3:  adjusted SHR  (95% CI) |
| **Acute phase** |  |  |  |  |  |  |
| Mild | Ref. | Ref. | Ref. | Ref. | Ref. | Ref. |
| Moderate | 3.68 (2.88, 4.71) | 3.64 (2.81, 4.70) | 3.53 (2.67, 4.66)^b^ | 5.21 (2.21, 12.26) | 5.30 (2.27, 12.36) | 5.41 (2.29, 12.77)^d^ |
| Severe | 5.32 (4.14, 6.85) | 5.19 (3.95, 6.82) | 5.45 (4.04, 7.35)^b^ | 3.57 (1.53, 8.30) | 3.82 (1.62, 9.04) | 4.06 (1.74, 9.51)^d^ |
| **Subacute phase** |  |  |  |  |  |  |
| Mild | Ref. | Ref. | Ref. | Ref. | Ref. | Ref. |
| Moderate | 3.02 (2.55, 3.58) | 3.04 (2.55, 3.63) | 2.89 (2.38, 3.52)^c^ | 3.04 (1.94, 4.77) | 3.05 (1.95, 4.78) | 3.09 (1.97, 4.85)^d^ |
| Severe | 4.22 (3.54, 5.04) | 4.25 (3.51, 5.14) | 4.56 (3.69, 5.63)^c^ | 1.87 (1.19, 2.93) | 1.93 (1.21, 3.06) | 2.02 (1.28, 3.19)^d^ |
| ^a^ Adjusted for age and sex.  ^b^ Adjusted for selected predictors: stroke severity, age, sex, active cancer, previous VTE, recent trauma or surgery, postmenopausal hormone therapy, educational level, cohabitation status, and smoking.  ^c^ Adjusted for selected predictors: for stroke severity, age, sex, active cancer, previous VTE, recent trauma or surgery, postmenopausal hormone therapy, educational level, and smoking.  ^d^ Adjusted for selected predictors: stroke severity, sex, active cancer, and previous VTE. | | | | | | |
